# The Flavonoid Luteolin Worsens Chemical-Induced Colitis in NF-κB^EGFP^ Transgenic Mice through Blockade of NF-κB-Dependent Protective Molecules

**DOI:** 10.1371/journal.pone.0000596

**Published:** 2007-07-04

**Authors:** Thomas Karrasch, Joo-Sung Kim, Byung Ik Jang, Christian Jobin

**Affiliations:** Department of Medicine and Center for GI Biology and Disease, University of North Carolina at Chapel Hill, Chapel Hill, North Carolina, United States of America; University of Cambridge, United Kingdom

## Abstract

**Background:**

The flavonoid luteolin has anti-inflammatory properties both in vivo and in vitro. However, the impact of luteolin on experimental models of colitis is unknown.

**Methodology/Principal Findings:**

To address the therapeutic impact of luteolin, NF-κB^EGFP^ transgenic mice were fed a chow diet containing 2% luteolin- or isoflavone-free control chow (AIN-76), and acute colitis was induced using 3% dextran sodium sulfate (DSS). Additionally, development of spontaneous colitis was evaluated in IL-10^−/−^;NF-κB^EGFP^ transgenic mice fed 2% luteolin chow diet or control chow diet. Interestingly, NF-κB^EGFP^ transgenic mice exposed to luteolin showed worse DSS-induced colitis (weight loss, histological scores) compared to control-fed mice, whereas spontaneous colitis in IL-10^−/−^;NF-κB^EGFP^ mice was significantly attenuated. Macroscopic imaging of live resected colon showed enhanced EGFP expression (NF-κB activity) in luteolin-fed mice as compared to control-fed animals after DSS exposure, while cecal EGFP expression was attenuated in luteolin-fed IL-10^−/−^ mice. Interestingly, confocal microscopy showed that EGFP positive cells were mostly located in the lamina propria and not in the epithelium. Caspase 3 activation was significantly enhanced whereas COX-2 gene expression was reduced in luteolin-fed, DSS-exposed NF-κB^EGFP^ transgenic mice as assessed by Western blot and immunohistochemical analysis. In vitro, luteolin sensitized colonic epithelial HT29 cells to TNFα-induced apoptosis, caspase 3 activation, DNA fragmentation and reduced TNFα-induced C-IAP1, C-IAP2 and COX-2 gene expression.

**Conclusions/Significance:**

We conclude that while luteolin shows beneficial effects on spontaneous colitis, it aggravates DSS-induced experimental colitis by blocking NF-κB-dependent protective molecules in enterocytes.

## Introduction

The gastrointestinal tract of higher organisms is lined by a single layer of intestinal epithelial cells. This physical barrier separates subepithelial mucosal immune cells such as lymphocytes and myeloid cells from a variety of antigenic substances present within the intestinal lumen (e.g. bacteria, bacterial products, food antigens) [1], [2]. Consequently, the integrity of the epithelial barrier is essential for the maintenance of host homeostasis, as it prevents a dysregulated uptake of luminal antigens. The incidence and prevalence of ulcerative colitis and Crohn's disease, collectively referred to as inflammatory bowel diseases (IBD), have been increasing in developed countries worldwide over the last few decades [3]. Of note, the knowledge of basic molecular and cellular mechanisms leading to IBD has significantly increased over the past decade [4]. For example, multiple studies support the concept that an improper activation of effector T cells in conjunction with insufficient regulatory T cell activity are key events leading to the development of IBD [5]. Interestingly, the endogenous intestinal flora itself seems to play an important role in initiating the dysregulated host immune response [6], leading to the release of a number of inflammatory mediators such as IL-1, IL-6, IL-12, IL-23, TNFα and IFNγ that participate in the pathology of the disease [7]. The transcription factor NF-κB controls the production of many of these inflammatory mediators, and we recently demonstrated its important role in driving bacteria-induced chronic intestinal inflammation [8]. Consequently, this transcriptional system represents a potential therapeutic target to treat IBD [9], [10].

Mainstream treatments to manage IBD are largely based on immunosuppressive approaches with broad acting agents such as prednisone, cyclosporin A and tacrolimus (FK506) [11]. Although these are relatively effective, a number of patients develop significant side effects and/or become unresponsive to them. These concerns and the perception that alternative medicine is “healthier” than classical therapeutic options lead a growing segment of the population to seek alternative treatments to ameliorate various disorders including chronic intestinal inflammation [12]. This is clearly exemplified by the considerable amount of money spent by the general population on alternative medicine with an estimated world market of 62 billion dollars [13], [14]. Herbal medicine encompassing extracts or active components derived from plants, barks, roots, leaves, flowers, and fruits represents an extremely popular segment of alternative medicine. However, despite their clear popularity, absence of empirical data showing efficacy and mechanisms of action in vivo prevents their incorporation into mainstream medicine.

Of interest, a large number of dietary products have been shown to inhibit NF-κB activity in different cell systems [15]. Luteolin for example is a flavonoid present in significant amounts in vegetables including celery, sage, carrots and broccoli, and a healthy diet is believed to contain between 2 mg and 125 mg of luteolin per day [16], [17]. We recently demonstrated that luteolin suppresses LPS-induced NF-κB signaling both in vivo and in vitro through inhibition of the IκB-kinase complex [18]. In addition, numerous other studies have shown the anti-inflammatory action of this flavonoid both in vivo and in vitro [19]–[24]. Consequently, this polyphenolic compound may have a beneficial impact either in treating IBD or preventing their development.

In the present study, we examined the impact of luteolin on the DSS model of acute colitis in mice. To follow patterns of NF-κB activation, we conducted experiments using NF-κB^EGFP^ and IL-10^−/−^;NF-κB^EGFP^ transgenic mice, which express the reporter gene enhanced green fluorescent protein (EGFP) under control of the NF-κB promoter. We found that luteolin significantly enhanced intestinal epithelial cell caspase-3 activation and prevented the induction of cytoprotective molecules such as COX-2 following DSS-exposure. DSS-exposed, luteolin-fed mice demonstrated more severe colitis than control-fed animals, whereas spontaneous colitis in IL-10^−/−^;NF-κB^EGFP^ mice was significantly attenuated. We conclude that while luteolin shows beneficial effects on spontaneous colitis, it aggravates DSS-induced experimental colitis by blocking NF-κB-dependent protective molecules in enterocytes.

## Materials and Methods

### DSS-induced acute colitis

The NF-κB^EGFP^ transgenic mice were described previously [25]. These mice allow the real-time assessment of NF-κB activity (EGFP expression) during various experimental treatments. Mice were maintained in standard housing cages in a specific pathogen free (SPF) environment and allowed to drink and feed ad libitum at all times. Luteolin (SYNORx; San Clemente, CA) was incorporated in standard laboratory chow AIN-76 in different amounts (weight-percentages: 0.5%, 2%, 5%) (Research Diets Inc; New Brunswick, NJ). Mice (7/group) were pre-fed luteolin-chow (0.5%, 2%, 5%) or control chow (AIN-76) for 3 days (loading period) as described previously [26], [27]. After this time, mice were given 3% DSS (MP Biomedicals; Aurora, OH) in drinking water for an additional 6 days. Control mice were given water. Water consumption was comparable between the different groups. Similarly, chow consumption (control AIN76 and luteolin) was comparable between DSS and water control groups, both before and during the induction of colitis (daily consumption approximately 1.6 g/mouse, equaling 32 mg luteolin; differences≤0.08 g/day or 5%). Mice were monitored daily for weight loss as well as signs of rectal bleeding (Hemoccult, Beckmann Coulter Inc; Fullerton, CA). At day 6 of DSS administration, mice were sacrificed, sections were taken from the distal colon for histological assessment of inflammation and EGFP-expression was imaged as described below.

### Spontaneous colitis in IL-10^−/−^;NF-κB^EGFP^ transgenic mice

IL-10^wt/wt^ and IL-10^−/−^ mice (Sv129 background) were cross-bred to NF-κB^EGFP^ mice (C57Bl/6 background), and IL-10^wt/wt^;NF-κB^EGFP^ and IL-10^−/−^;NF-κB^EGFP^ mice generated in the F2-generation were derived into germ-free condition. Germ-free IL-10^wt/wt^;NF-κB^EGFP^ and IL-10^−/−^;NF-κB^EGFP^ littermates were transferred to a specific-pathogen-free (SPF) environment and immediately placed on a diet containing 2%-luteolin versus AIN-76-control-chow (7/group). After 4 weeks, mice were sacrificed, sections were taken from cecum and distal colon for histological assessment of inflammation and EGFP-expression was imaged as described below. All animal experiments were approved by the Institutional Animal Care and Use Committee of the University of North Carolina at Chapel Hill.

### Sample collection and histological evaluation

Mice were sacrificed at designated time-points by CO_2_ asphyxiation. The entire colon was dissected and flushed with ice-cold PBS. Colonic cross-sections were taken, fixed in 10% formalin for 24 h and embedded in paraffin to provide sections for histological evaluation. Severity of colitis was evaluated in Hematoxylin-Eosin-stained sections by an experienced investigator blinded to the experimental conditions. For DSS-induced colitis, the distal colon was evaluated because it has been demonstrated to be the most severely affected colonic segment in DSS-induced colitis [28], [29] and to provide a score representing the disease severity in the entire colon [30]. Coded sections were scored using a validated scoring system developed by Cooper [29] and Dieleman [31], and modified by Williams [30] using a scale of 0 to 40. For each animal, 2 sections approximately 400 µm apart were scored and averaged. For spontaneous colitis in IL-10^−/−^ mice, mucosal inflammation was evaluated in cross-sections of the cecum and the distal colon separately. Sections were scored in a blinded fashion on a scale from 0 to 4, based on the degree of lamina propria mononuclear cell infiltration, crypt hyperplasia, goblet cell depletion and architectural distortion, as previously described [32].

### Immunohistochemical evaluation

Colonic sections were prepared as described above. Immunohistochemical staining for activated caspase 3 (antibodies to cleaved caspase-3 (Asp175); Cell Signaling Technology Inc; Beverly, MA) and cyclooxygenase 2 (COX-2) (Cayman Chemical; Ann Arbor, MI) was performed according to the manufacturer's directions. The sections were counterstained with a mixture of methyl-green and alcian-blue. Two different sections (per animal) approximately 400 µm apart were evaluated, and representative sections are shown.

### Primary intestinal epithelial cell isolation

Primary intestinal epithelial cells were isolated as described previously [33]. Briefly, colons were opened longitudinally, and colonic intestinal epithelial cells were separated by shaking the colons in a solution containing 3 mM EDTA and 0.5 mM DTT for 90 min. The remaining tissue was discarded and epithelial cells in the supernatant were spun down at 1000 rpm for 5 min. The cell pellet was then directly lysed in 1× Laemmli buffer. Vimentin or CD64, markers for myofibroblasts and myeloid cells respectively, were not detected, whereas the epithelial marker cytokeratin was strongly expressed in our epithelial cell preparation as assayed by Western blot analysis (data not shown).

### HT29 cell culture, stimulation, cell and whole colonic tissue protein isolation

Human transformed colonic epithelial HT29 cells (passage 20–30) (American Type Culture Collection (ATCC); Manassas, VA) were cultured as described previously [34]. Before experiments, cells were starved overnight in serum-reduced media (1% serum). Where indicated, HT29 cells were treated with luteolin (SYNORx; San Clemente, CA) dissolved in DMSO, TNFα (R&D Systems; Minneapolis, MN) or solvent control for designated times, respectively. DMSO alone had no biological effect on the various in vitro readouts (data not shown). Stimulated or control treated HT29 cells were collected and directly lysed in 1X Laemmli buffer.

The colons from mice in the various groups were dissected, flushed with ice-cold PBS and homogenized for 15 s with a polytron (IKA Works Inc; Wilmington, NC) in a solution containing 50 mM Hepes, 150 mM NaCl, 20 mM NaPyrophosphate, 100 mM NaF, 1% Triton X-100, 10 mM EDTA and 1X Proteinase inhibitors (Complete Mini, Roche Diagnostics GmbH; Penzberg, Germany). After incubation at 4°C for 30 min with gentle agitation, undissolved contents were spun down and the supernatant mixed 1∶1 with 2× Laemmli buffer.

### Western Blot analysis

The protein concentration of lysates was measured using Bio-Rad quantification assay (Bio-Rad Laboratories; Hercules, CA). Proteins (20 µg) were separated using 10% (13% for caspase analysis) SDS-PAGE and transferred to nitrocellulose membranes. Antibodies to COX-2 (Cayman Chemicals; Ann Arbor, MI), cleaved caspases 3, 8 and 9 (Cell Signaling Technology Inc, Beverly, MA) and β-actin (ICN; Costa Mesa, CA) were used at a dilution of 1:1000 in a solution containing 5% milk in TBS-Tween (0.1%). Immunoreactive proteins were detected using the enhanced chemiluminescence light (ECL) detecting kit (Amersham Biosciences, Piscataway, NJ) as described previously [35].

### Macroscopic and confocal assessment of EGFP expression

Mice were sacrificed at designated time points, the entire colon dissected and then directly imaged for EGFP expression in a light-tight imaging box without further preparation using a CCD camera and emission filters specific for EGFP (Lightools Research; Encinitas, CA).

For confocal microscopy, the colons were opened longitudinally and placed on the stage of a Leica SP2 Upright Laser Scanning Confocal Microscope (Leica, Germany), lumen side facing the objective lens without further processing or fixation. EGFP was excited with a 495 nm laser, and images were acquired using detection filters specific for the EGFP emission spectrum. Images were overlayed with those obtained using transmitted light, allowing for spacial orientation relative to the shadows of the crypts. Images were analyzed using the Leica SP2 Laser Scanning Confocal Software (Leica, Germany).

### PGE_2_ secretion

Luteolin-treated cells were stimulated with TNFα (5 ng/ml) for 24 h. A PGE_2_ enzyme linked immunosorbent assay (ELISA) of cell culture supernatants was performed in triplicate according to the manufacturer's instructions (Assay Designs; Ann Arbor, MI).

### RNA extraction and amplification by RT-PCR

RNA was isolated using the TRIzol method (Invitrogen; Carlsbad, CA), reverse transcribed (1 µg RNA), and amplified as previously described [35] using specific primers for mouse C-IAP1, C-IAP2, X-IAP, COX-2 and β-actin. The PCR products were separated on 2% agarose gels containing gel Star fluorescent dye (FMC; Philadelphia, PA). Fluorescence staining was captured using an Alpha Imager 2000 (AlphaInnotech; San Leandro, CA). Sequences of primers were 5′-CAATTCGGCACCATAACTCTG-3′ and 5′-CCTGTGGTTAAATCTGCCTTG-3′ for human C-IAP1 (amplicon length 1067 pb), 5′-CAAGTAGATGAGGGTAACTGGC-3′ and 5′-AAGTTCCATCCCCTGTCCAATG-3′ for human C-IAP2 (amplicon length 550 pb), 5′-CTTGCATACTGTCTTTCTGAGC-3′ and 5′-ACACCATATACCCGAGGAAC-3′ for human X-IAP (amplicon length 818 pb) [36], 5′-AGATCATCTCTGCCTGAGTATCTT-3′ and 5′-TTCAAATGAGATTGTGGGAAAATTGCT-3′ for human COX-2 (amplicon length 304 pb) and 5′-CCAACCGCGAGAAGATGACC-3′ and 5′-GATCTTCATGAGGTAGTCAGT-3′ for human β-actin (amplicon length 235 pb).

### DNA fragmentation analysis and apoptosis detection

HT29 cells were grown in six well plates and at 80% of confluence were treated with various amounts of luteolin and then stimulated with TNFα (20 ng/ml) for 6, 12 and 24 hours. Cells were washed twice with PBS and DNA was extracted with a hypotonic lysis buffer (10 mM Tris, 1 mM EDTA and 0,2% Triton X-100, ph 7.5). Proteinase K digestion was carried out for 16 h at 37°C. DNA was then extracted with phenol-chlorophorm-isoamyl-alcohol (25∶24∶1). After ethanol precipitation and resuspension in 10 mM Tris, 1mM EDTA, ph 8.0, the DNA was electrophoresed on a 1.5% agarose gel. A 100 bp DNA ladder (Life Technologies, Rockville, MD) was used as a molecular weight standard. DNA fragmentation was quantified by detecting histone-associated DNA fragments in enzyme linked immunoabsorbance assay (ELISA) (Boehringer Mannheim; Indianapolis, IN) according to the manufacturer's instructions.

### Caspase Activity and Inhibition

Cells were pretreated with luteolin and then stimulated with TNFα as indicated above. Cells were harvested, washed twice with PBS and then lysed in Apopain Lysis Buffer (10 mM HEPES, ph 7.4, 2 mM EDTA, 0.1% CHAPS, 5 mM DTT, 1mM PMSF, 10 µg/ml pepstatin A, 10 µg/ml aprotinin, 20 µg/ml leupeptin). Lysates were cleared by centrifugation for 30 min at 12000 rpm in a microfuge at 4°C. Protein content was measured using the Bio-Rad protein quantification assay (Bio-Rad; Hercules, CA). Caspase-3 activity was monitored in vitro using the fluorogenic peptide substrate Z-DEVD-AFC (Bio-Rad; Hercules, CA). Fluorescence changes derived from free AFC released by caspase-3 activity were detected at 500–550 nm with a GeminiX Spectrafluorometer (Molecular Devices; Sunnyvale, CA). Results are presented in fold changes.

### Luciferase adenoviral vector infection and luciferase activity assay

HT29 cells were infected for 16 h with an adenoviral vector encoding a NF-κB-luciferase reporter gene (Ad5κB-LUC) as described previously [34]. The Ad5GFP containing GFP was used as a viral vector infection negative control. The cells were washed, and fresh medium containing serum was added. Cells were then pretreated with various concentrations of luteolin or solvent control for 45 min, after which time they were stimulated with TNFα (5 ng/ml) for 12 h. Cell extracts were prepared using luciferase cell lysis buffer (PharMingen; San Diego, CA) according to the manufacturer's instructions. Luciferase activity was quantified using luciferase substrate solutions A and B from PharMingen and read on a Lmax luminometer microplate reader (Moleculer Devices; Sunnyvale, CA) and results were normalized to protein concentration measured with the Bio-Rad protein quantification assay (Bio-Rad; Hercules, CA).

### Intestinal epithelial cell restitution studies in vitro

Restitution was assessed in rat non-transformed ileal epithelial cells IEC-18 as previously described [37]–[39]. Briefly, IEC-18 cells were grown to confluency in 100 mm cell culture dishes, starved in serum-reduced media (1%) overnight and standard linear wounds were created using a sterile razor-blade. Cells were then washed once with PBS, and serum-reduced media was replenished. Restitution was calculated as migration over the wound-edge over 24 hours.

### Statistical analysis

Data are expressed as means±S.D. Groups of data (histological scores, body weight) were analyzed using Kruskal-Wallis non-parametric test and if the result indicated statistical differences (p<0.05) among groups, comparisons between groups were conducted using Mann-Whitney U test. Differences were considered significant if p<0.05. In vitro experiments were conducted in triplicates; representative results are shown. For in vitro experiments, data were analyzed by non-parametric *t*-test or Wilcoxon rank sum test where appropriate. Differences were considered significant if 2-tailed p values were <0.05.

## Results

### Luteolin-treatment worsens DSS-induced experimental colitis

**Figure 1:**
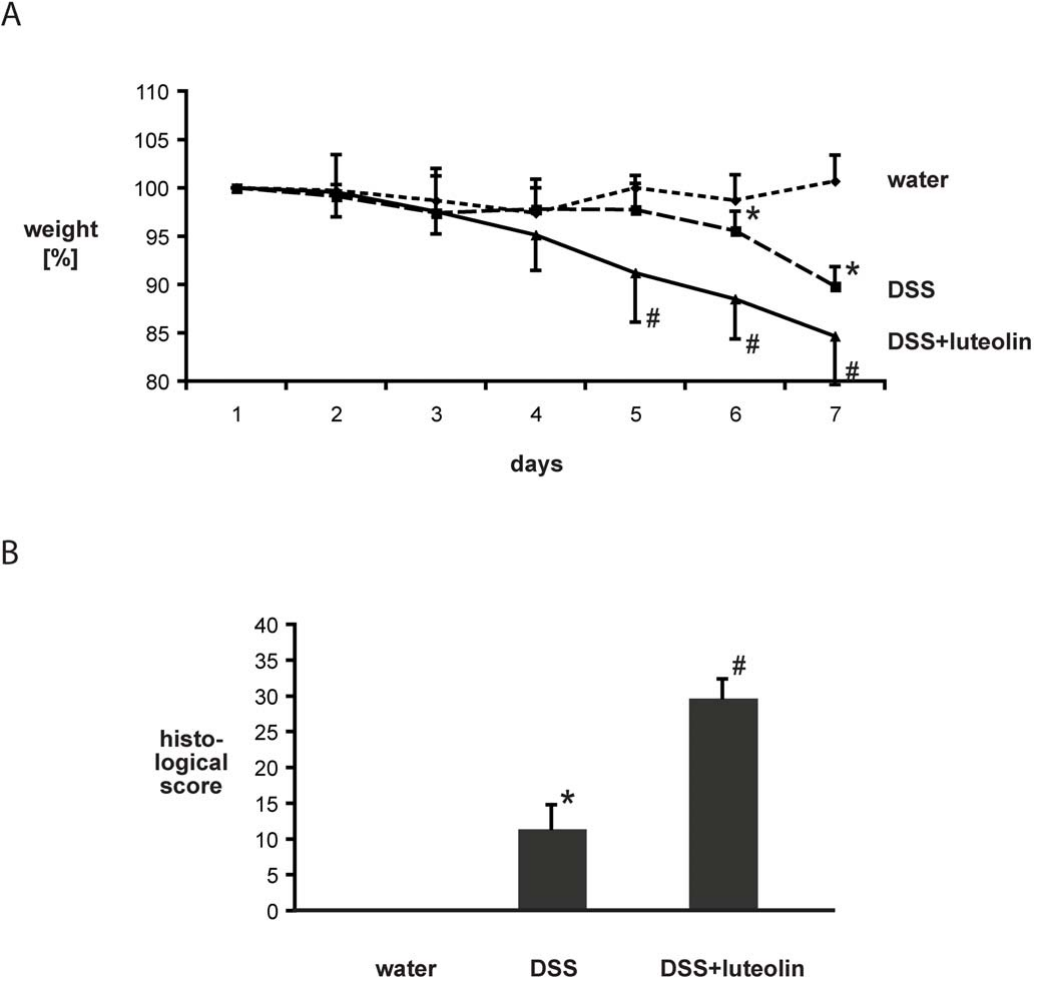
2% luteolin diet worsens DSS-induced experimental colitis as measured by weight loss and histologic score. 2% luteolin or AIN-76 fed mice (n = 7 per group) were exposed to 3% DSS in drinking water ad libitum for the indicated time and monitored as described in the Methods section. Weight loss in response to DSS is presented as percent of the starting weight (* p<0.05 versus control, # p<0.05 versus DSS alone) (A). Colonic histological sections were scored as described in the Methods section (* p<0.05 versus control, # p<0.05 versus DSS alone) (B).

Although the anti-inflammatory potential of the flavonoid luteolin has been established in vitro [18], [21]–[23], its therapeutic impact on different inflammatory disorders is still unclear. We tested the effect of luteolin in a murine model of chemical-induced acute intestinal inflammation. An NF-κB^EGFP^ transgenic mouse recently generated by our group allows the assessment of the kinetics and cellular localization of NF-κB-induced transcription (EGFP expression) during an inflammatory response [25]. NF-κB^EGFP^ mice (C57Bl/6; 7/group) were fed an isoflavone-free (AIN-76) or 2% luteolin diet for 3 days before being exposed to 3% DSS in drinking water ad libitum or tap water control. Mice were then sacrificed at day 6 and inflammation evaluated using established clinical and histological parameters [30], [31]. Surprisingly, although luteolin has systemic anti-inflammatory properties in vivo [18], luteolin-fed animals lost more weight than control diet-fed mice (8.8% versus 2.3% at day 4, 15.4% versus 10.2% at day 6, p<0.05) (Fig. 1A). Moreover, histological evaluation demonstrated significantly worse colitis in mice fed the luteolin chow compared to the AIN-76 control chow (Fig. 1B). Colitis was also exacerbated in mice fed a 0.5% or a 5% luteolin rich diet compared to AIN-76 control diet (data not shown). Mice fed with luteolin chow alone and exposed to water did not develop any signs of colitis (data not shown). These findings demonstrate that the flavonoid luteolin worsens DSS-induced colitis in mice.

### Luteolin-fed, DSS exposed NF-κB^EGFP^ mice display strong EGFP expression in the intestine

**Figure 2:**
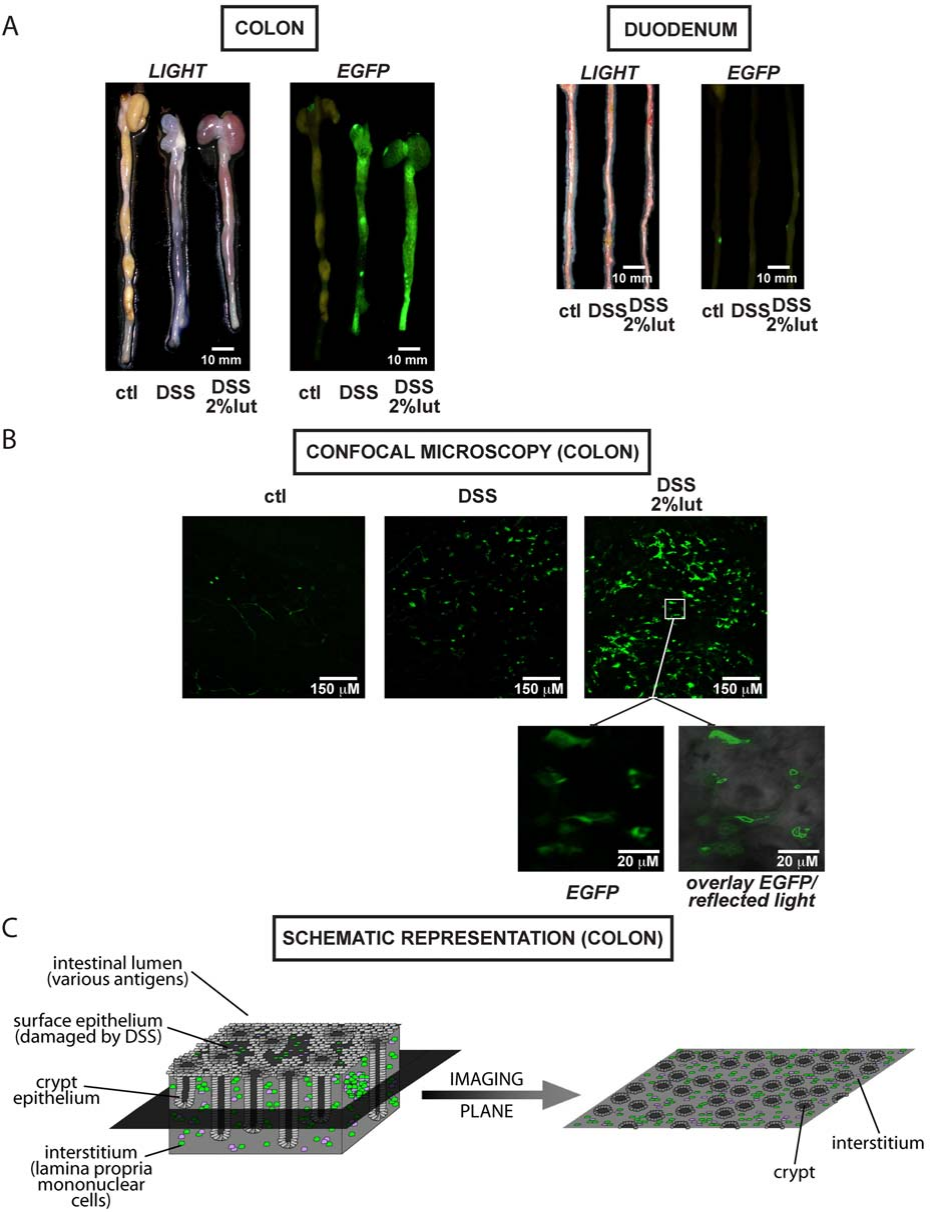
Enhanced EGFP expression in luteolin fed, DSS exposed NF-κB^EGFP^ mice as compared to control diet is confined to lamina propria mononuclear cells. NF-κB^EGFP^ mice (n = 7 per group) were sacrificed after 6 days of 3% DSS exposure, and dissected colon (left panel) and duodenum/proximal jejunum (right panel) were imaged at this time for EGFP using the Lightools Research macroimaging system with a dual output lighting (right). Photomicrographs (Nikon cool-pix digital camera) are shown on the left. Representative images are shown (A). Confocal imaging of colonic sections from NF-κB^EGFP^ mice (n = 3 per group) after 6 days of 3% DSS exposure was performed immediately after dissection as described in the Methods. Representative results are shown (B). A schematic representation of the colon indicates the plane depicted in the confocal microscopic images. Importantly, EGFP accumulation (NF-κB activation) is predominantly seen in lamina propria mononuclear cells located in between the crypts (LPMNC, light purple and green), and not in surface and crypt epithelium, which is damaged by DSS (light grey) (C).

To gain insight into the molecular mechanisms involved in the worsening of colitis in luteolin-fed mice, we investigated the in vivo pattern of NF-κB activity. The colons of 2% luteolin or AIN-76 fed DSS-treated NF-κB^EGFP^ mice were dissected and imaged using a CCD camera in a light-tight imaging box with a dual filtered light source and emission filters specific for EGFP. As seen in Fig. 2A, EGFP expression is strongly enhanced throughout the colon of DSS-exposed animals compared to water-control mice (left panel). Of note, there was no difference in the macroscopic EGFP expression in duodenal and proximal jejunal sections of the same mice (Fig. 2A; right panel). This suggests that enhanced colonic EGFP expression correlates with DSS-induced colonic damage. Interestingly, colonic EGFP expression was not blocked in mice fed the 2%-luteolin diet, but rather showed enhanced expression, especially in the distal colon (Fig. 2A).

To examine the expression of EGFP at the cellular level, we visualized the distribution of the EGFP-positive cells present in colonic segments freshly harvested from NF-κB^EGFP ^mice using confocal microscopy. As seen in Fig. 2B, EGFP expression was increased in colons from mice fed the 2%-luteolin diet compared to those from untreated, DSS-exposed mice. Higher magnification images demonstrated that the increased EGFP expression was confined to interstitial mononuclear cells between crypts (Fig. 2B; schematic representation shown in Fig. 2C). These findings indicate that luteolin enhances DSS-induced colitis, which correlates with increased NF-κB activation (EGFP expression) from colonic mucosa lamina propria mononuclear cells (LPMNC).

### Decreased COX-2 gene expression and enhanced caspase 3 activation in luteolin fed mice following DSS exposure

DSS-induced colitis is exacerbated in COX-2 gene deficient mice [40], and a recent report demonstrated the importance of enterocyte-derived COX-2 gene expression for the amelioration of DSS-induced epithelial damage [41]. Since we previously showed that luteolin prevents NF-κB activation in enterocytes [18], we next investigated whether intestinal epithelial COX-2 expression is down-regulated in luteolin-fed mice.

**Figure 3:**
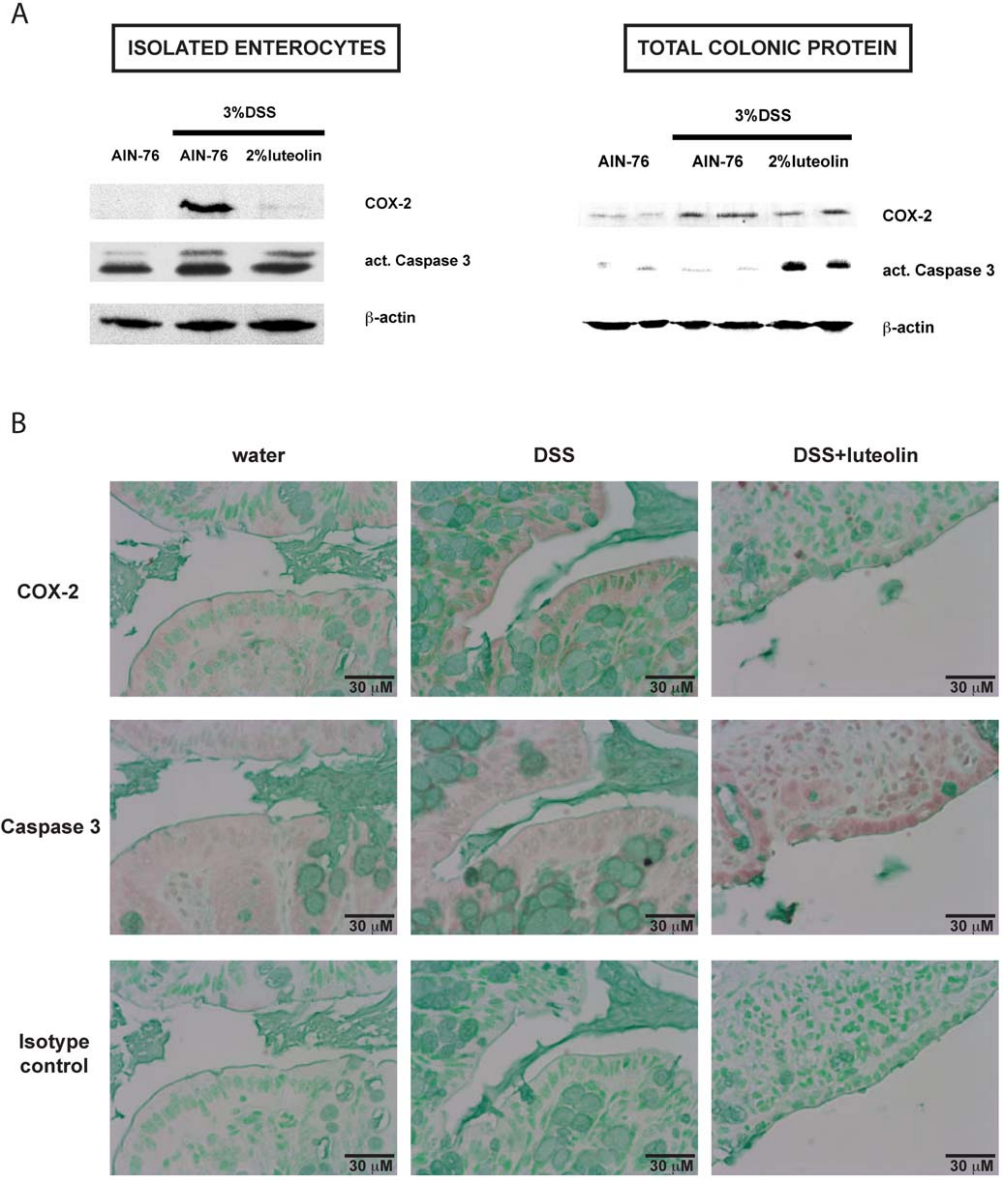
2% luteolin enhances caspase 3 activation and suppresses COX-2 protein accumulation in enterocytes after DSS exposure. NF-κB^EGFP^ mice (n = 7/group) were sacrificed after 2 days of 3% DSS exposure and protein (20 µg) from primary enterocytes (A, left) and total colonic lysates (A, right) were subjected to SDS-PAGE. COX-2 expression and caspase 3 processing were evaluated using specific antibodies against cleaved caspase 3 and COX-2, respectively, and immunoreactive proteins were detected using the enhanced chemiluminescence (ECL) technique (A). NF-κB^EGFP^ mice (n = 7/group) were sacrificed after 2 days of 3% DSS exposure, and cellular localization of COX-2 proteins (top panel) and caspase 3 processing (middle panel) were analyzed in the colon using IHC. Sections were counterstained with a mixture of methyl-green and alcian-blue. An isotype-control antibody was used to determine staining specificity on the same colonic section (lower panel). Representative results out of 2 different sections per each animal (n = 14/group) are shown (B).

Molecular alterations likely precede clinical signs of colitis and histopathological evidence of inflammation that generally occur after 3–4 days of DSS exposure in mice (Fig. 1A and data not shown). Therefore, we examined COX-2 expression by Western blot in enterocytes isolated from AIN-76-chow or 2%-luteolin-chow fed mice exposed to 3% DSS for only 2 days. Interestingly, COX-2 protein levels were higher in enterocytes isolated from DSS-exposed mice compared to those isolated from controls, and this effect was abrogated by luteolin treatment (Fig. 3A, left). A similar though less pronounced effect on COX-2 gene expression could be observed in total colonic protein extracts (Fig. 3A, right). Immunohistochemistry also demonstrated a strong decrease of DSS-induced COX-2 expression in enterocytes in mice fed the 2%-luteolin-chow (Fig. 3B; top panel). Isotype antibody control demonstrated the specificity of the staining (Fig. 3B; bottom panel). These results suggest that luteolin interferes with DSS-induced enterocyte-derived COX-2 gene expression.

NF-κB signaling controls numerous cellular events including cell survival through induction of anti-apoptotic genes [42], [43]. We next investigated whether luteolin would inhibit anti-apoptotic signaling in enterocytes, a process that would enhance intestinal epithelial damage by DSS. Interestingly, a small increase in caspase 3 processing was observed in isolated enterocytes (Fig. 3A, left), although baseline processing is strongly increased in these cells compared to total colonic lysates. This is likely due to cell detachment-induced apoptosis (anoikis) caused by the isolation procedure [44]. Importantly, total colonic protein obtained from luteolin-fed DSS-treated mice displayed a significant increase in caspase 3 processing compared to AIN-76-fed and DSS-treated or water control treated mice (Fig. 3A, right). Furthermore, enhanced caspase 3 processing is detected in enterocytes from luteolin-fed, DSS-exposed mice compared to control diet as determined by immunohistochemical analysis (Fig. 3B; middle panel). These results suggest that luteolin increases enterocyte caspase 3 activation after DSS-exposure.

### Luteolin-treatment ameliorates spontaneous Th1-dependent colitis and EGFP expression in IL-10^−/−^; NF-κB^EGFP^ mice

**Figure 4:**
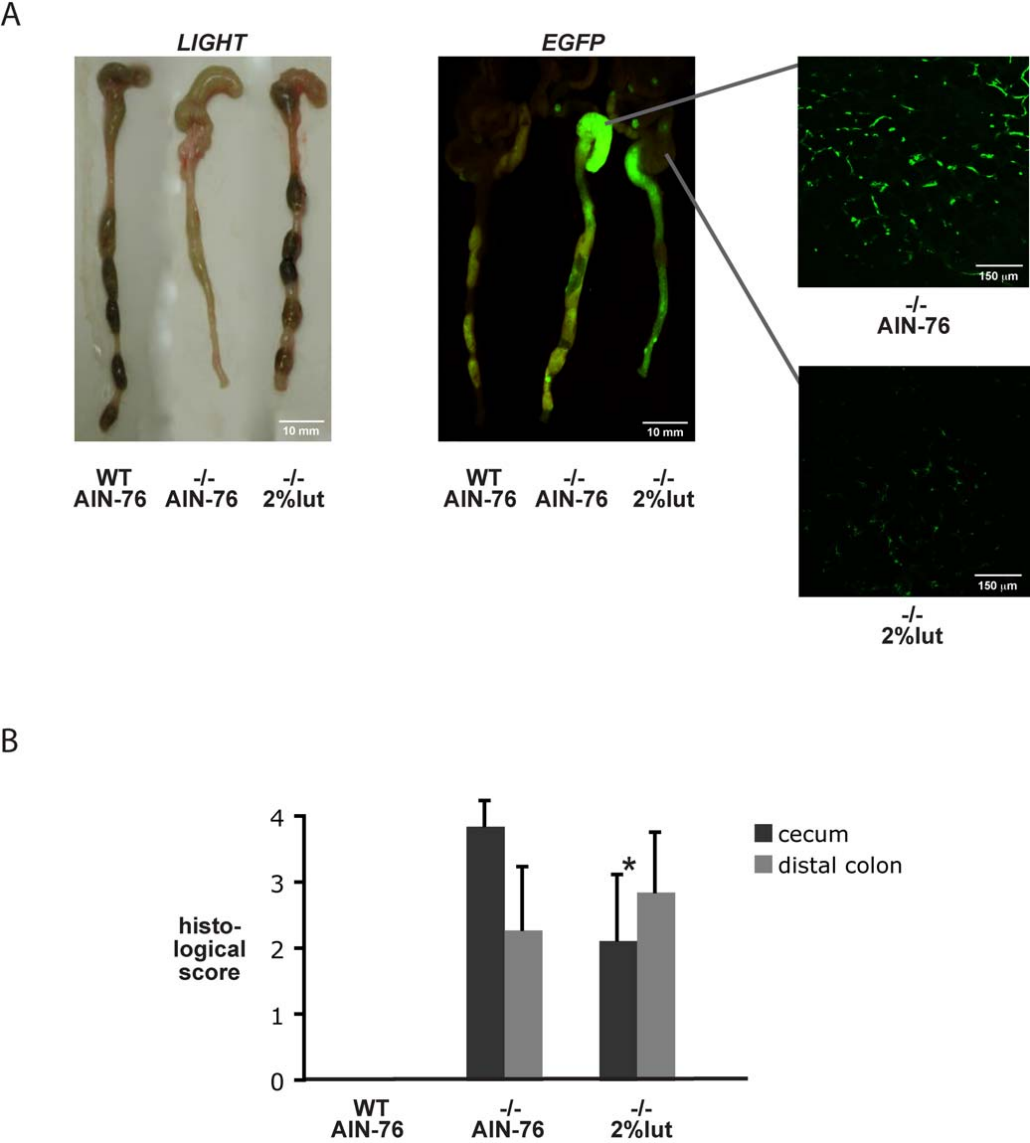
2% luteolin diet ameliorates cecal colitis and decreases lamina-propria EGFP expression in SPF-associated IL-10^−/−^;NF-κB^EGFP^ mice. Germ-free IL-10^wt/wt^;NF-κB^EGFP^ and IL-10^−/−^;NF-κB^EGFP^ mice were transferred to a specific-pathogen-free (SPF) environment and immediately placed on a diet containing 2% luteolin versus AIN-76 control chow (7/subgroup). After 4 weeks, mice were euthanized, and colonic EGFP-expression was imaged macroscopically and by confocal microscopy as described in figure 2 (A). Colonic histological sections from 4 weeks SPF-associated IL-10^wt/wt^;NF-κB^EGFP^ and IL-10^−/−^;NF-κB^EGFP^ mice were taken and scored as described in the Methods section (* p<0.05 versus AIN-76 control chow) (B).

We next asked if the NF-κB-inhibiting properties of luteolin might be beneficial in a spontaneous model of chronic, Th1-mediated intestinal inflammation. Germ-free IL-10^wt/wt^;NF-κB^EGFP^ and IL-10^−/−^;NF-κB^EGFP^ mice were transferred to a specific-pathogen-free (SPF) environment and immediately placed on a diet containing 2% luteolin versus AIN-76 control-chow (7/group). After 4 weeks, mice were sacrificed, sections were taken from cecum and distal colon for histological assessment of inflammation and EGFP expression was imaged as described above. Interestingly, IL-10^−/−^ mice fed with the control diet showed thickening of the colon and absence of solid stool compared to wild-type mice, whereas luteolin-fed IL-10^−/−^ mice displayed a macroscopically healthy colon with abundant and well-formed stool pellets (Fig. 4A). Furthermore, luteolin-treatment decreased cecal EGFP expression in IL-10^−/−^;NF-κB^EGFP^ mice (Fig. 4A). Histological assessment of inflammation showed that luteolin treatment ameliorated cecal inflammation in IL-10^−/−^;NF-κB^EGFP^ mice (Fig. 4B). Distal colonic inflammation and EGFP expression were comparable between luteolin and AIN76 control-fed mice (Fig. 4A,B). Thus, these results do not demonstrate adverse effects of luteolin in IL-10^−/−^ mice, but rather suggest a potential beneficial impact of this flavonoid in a model of spontaneous chronic T cell-mediated colitis.

### Luteolin-treatment sensitizes human colonic intestinal epithelial cells HT29 to TNFα-induced apoptosis

**Figure 5:**
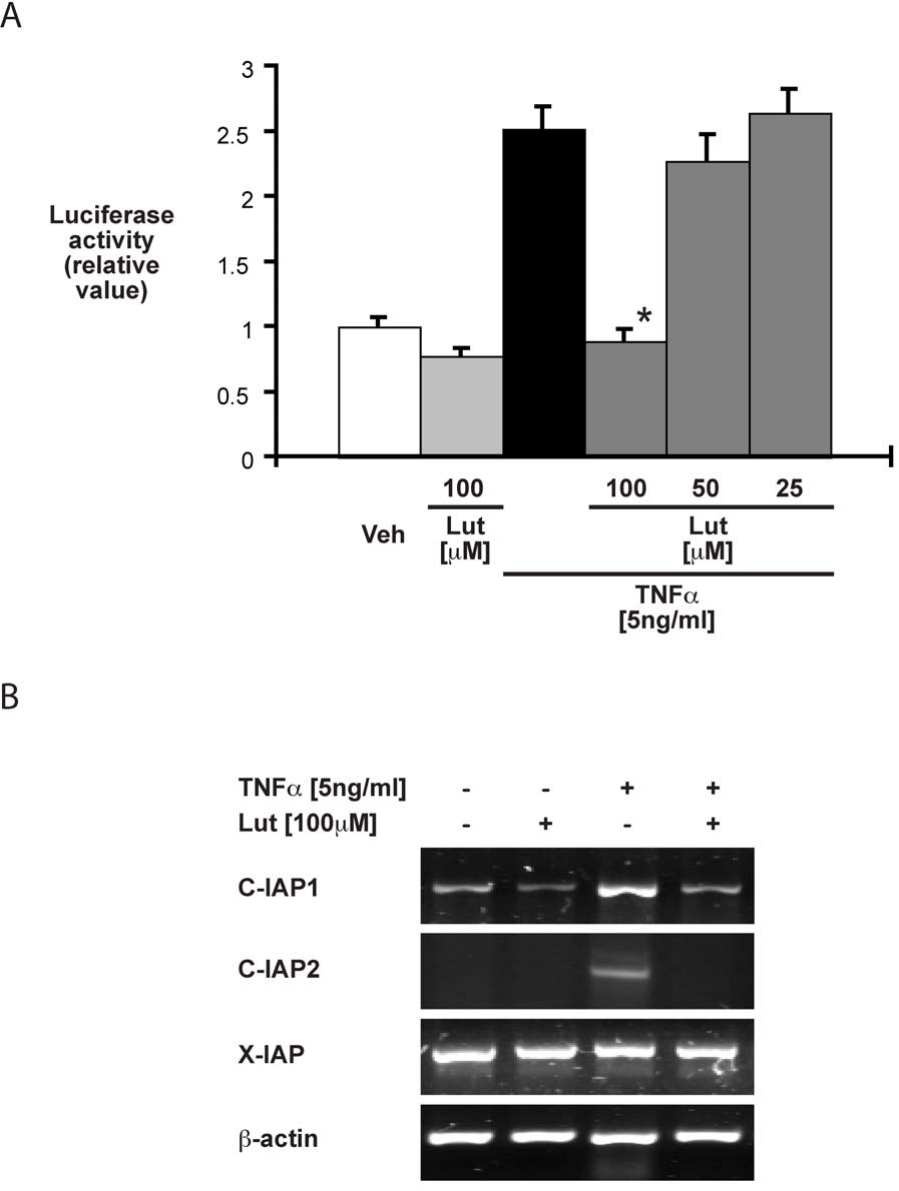
Luteolin blocks TNFα-induced NF-κB activity and suppresses TNFα-induced C-IAP1 and C-IAP2 mRNA accumulation in HT29 cells. HT29 cells were infected with Ad5κB-LUC, and TNFα (5 ng/ml) was added in the presence or absence of various concentrations of luteolin. Luciferase activity after 16h was measured using an Lmax microplate reader and results were normalized for extract protein concentrations. Representative results of at least 3 independent experiments are shown (* p<0.01 versus TNFα alone) (A). HT29 cells were pretreated with luteolin (100 µM) for 45min, and then stimulated with TNFα (5 ng/ml) for 4 h. Total RNA (1 µg) was extracted, reverse-transcribed, and amplified using specific human C-IAP1, C-IAP2, X-IAP and β-actin primers. Results are representative of three independent experiments (B).

We next characterized the impact of luteolin on TNFα-induced NF-κB activity, COX-2 and anti-apoptotic gene expression in vitro, using the human colon epithelial cell line HT29. Cells were infected for 16 h with an adenoviral vector encoding an NF-κB-luciferase reporter gene (Ad5κB-LUC), pretreated with various doses of luteolin (25 µM, 50 µM or 100 µM) for 45min and then stimulated with TNFα (5 ng/ml) for 12 h. As shown in Fig. 5A, luteolin (100 µM) inhibited TNFα-induced NF-κB transcriptional activity in HT29 cells. NF-κB-dependent anti-apoptotic genes include the cellular inhibitors of apoptosis protein-1 and -2 (C-IAP1 and C-IAP2), which selectively block the key pro-apoptotic enzymes caspase-3, -8 and -9. Interestingly, luteolin treatment completely suppressed TNFα-induced C-IAP1 and C-IAP2 mRNA accumulation, whereas X-IAP mRNA levels remained unchanged as measured by RT-PCR (Fig. 5B). Thus, luteolin prevents TNFα-induced NF-κB activity and anti-apoptotic gene induction.

**Figure 6:**
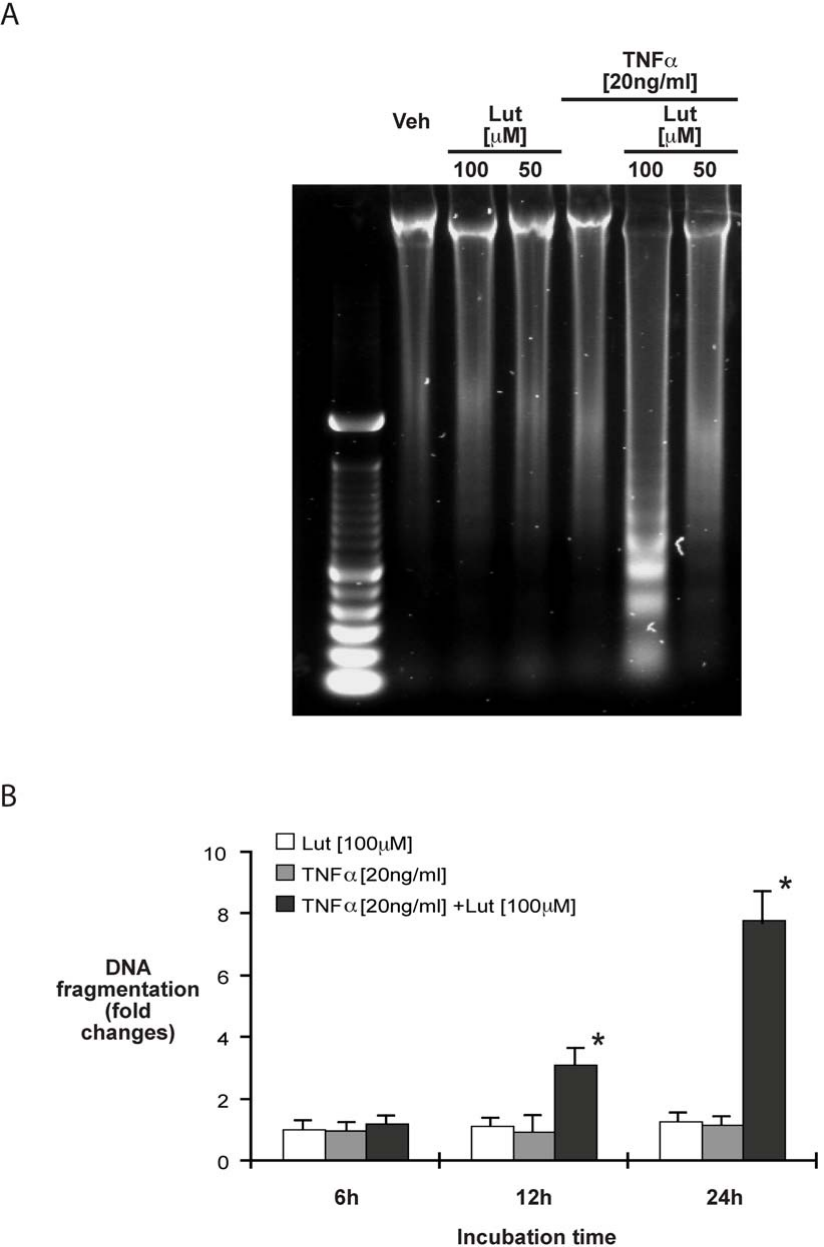
Luteolin-treatment sensitizes TNFα-exposed HT29 cells to DNA fragmentation and cytoplasmic accumulation of oligonucleosome-bound DNA. HT29 cells were pretreated with various concentration of luteolin alone or in combination with TNFα (20 ng/ml) for 24 h. Cells were lysed, DNA extracted and separated on a 1% agarose gel. Results are representative of three independent experiments (A). HT29 cells were treated as described above for 12 and 24 h. Cells were lysed and apoptosis measured using an ELISA-based assay as described in the Method section. Representative results of at least 3 independent experiments are shown (* p<0.01 versus TNFα alone) (B).

We next asked whether luteolin functionally impacts on HT29 cell apoptosis in the presence of TNFα. Cells were pretreated with luteolin (50 µM or 100 µM) for 45 min, and then exposed to TNFα (20 ng/ml) for 24 h, after which time genomic DNA was isolated. As seen in Fig. 6A, luteolin-treatment (100 µM) led to TNFα-induced DNA fragmentation in HT29 cells. Quantitative assessment of DNA fragmentation by ELISA assay for cytoplasmic oligonucleosome-bound DNA demonstrated a 2.2fold induction of DNA fragmentation in luteolin-pretreated TNFα stimulated cells by 12 h, which increased to 8.1fold by 24 h post-treatment (Fig. 6B). Thus, luteolin treatment leads to TNFα-induced DNA fragmentation in HT29 cells in vitro.

**Figure 7:**
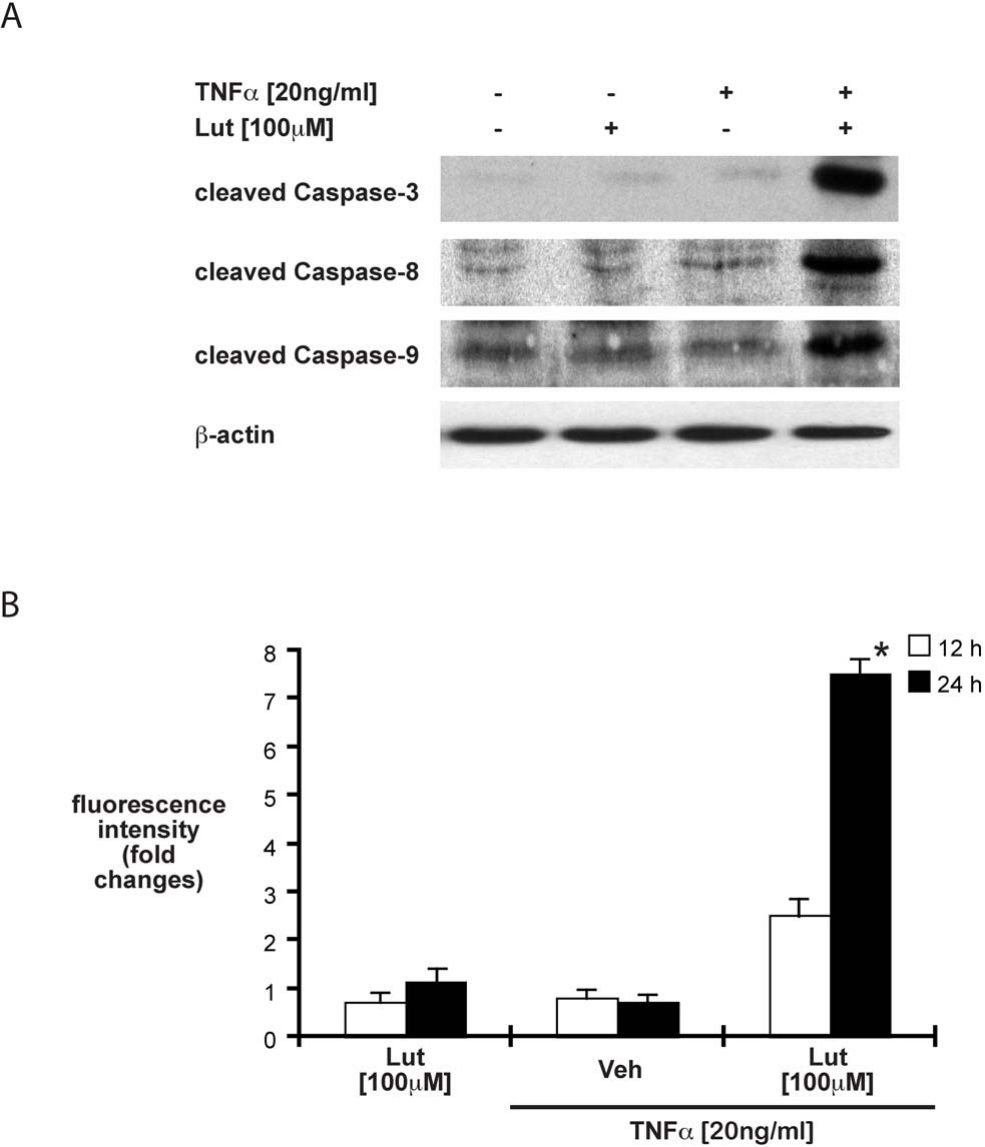
Luteolin-treatment induces caspase 3, 8 and 9 activation in the presence of TNFα in HT29 cells. HT29 cells were treated with luteolin (100 µM) alone or in combination with TNFα (20 ng/ml) for 12 h. Cells were lysed, protein extracts (20 µg) were subjected to SDS-PAGE (13%) and caspase 3, 8 and 9 processing was evaluated by Western blot using specific antibodies for cleaved caspases 3, 8 and 9. Immunoreactive proteins were detected using the enhanced chemiluminescence (ECL) technique. Representative results of at least 3 independent experiments are shown (A). Cells were treated as described above for 12 and 24 h. Cells were lysed and caspase 3 activity was quantified using a fluorescence-based assay as described in the Method section. Representative results of at least 3 independent experiments are shown (* p<0.01 versus TNFα alone at 24 h) (B).

Caspase-3 processing is enhanced in luteolin-fed mice exposed to DSS (Fig. 3A,B). We next investigated the impact of this flavonoid on caspase activity in the presence of TNFα. HT29 cells were treated with luteolin (100 µM) for 45 min, then treated with TNFα (20 ng/ml) for 24 h and caspase processing was determined by Western blot analysis. As shown in Fig. 7A, luteolin strongly induced caspase-3, -8 and -9 processing in the presence of TNFα. Moreover, luteolin induced caspase-3 activity in the presence of TNFα as demonstrated by a fluorescence-based assay (Fig. 7B; 2.5 fold after 12 h treatment, 7.5 fold after 24 h treatment). The kinetics of caspase activation parallel those of DNA fragmentation (Fig. 6B). In summary, luteolin blocks TNFα-induced NF-κB transcriptional activity and anti-apoptotic gene expression, but induces caspase-3, -8 and -9 processing in HT29 cells in the presence of TNFα in vitro.

### Luteolin-treatment suppresses TNFα-induced COX-2 gene expression

**Figure 8:**
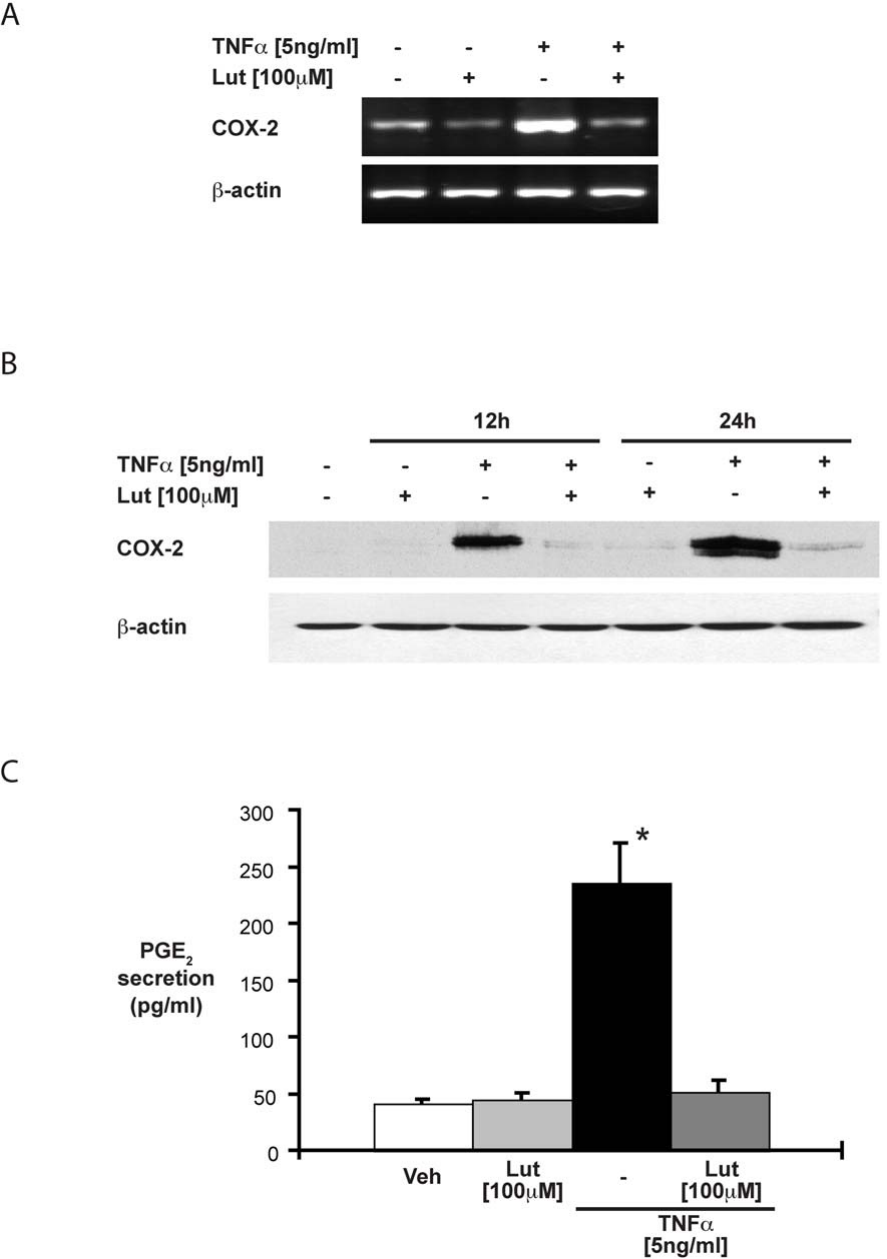
Luteolin-treatment inhibits TNFα-induced COX-2 gene expression in HT29 cells. HT29 cells were pretreated with luteolin (100 µM) for 45 min, and then stimulated with TNFα (5 ng/ml) for 4 h. Total RNA (1 µg) was extracted, reverse-transcribed, and COX-2 mRNA was amplified using specific human primers (A). Cells were treated as described above for 12 h and 24 h, and COX-2 protein accumulation was detected using Western blot analysis (B). Cells were treated as described above for 24 h and PGE_2_ production was measured from the cell culture supernatants using ELISA (* p<0.01 versus TNFα plus luteolin) (C). All data are representative of 3 independent experiments.

The NF-κB-dependent COX-2 gene has been associated with a protective host response during DSS-induced colitis [40], [41]. In addition, COX-2 gene expression has been shown to promote intestinal epithelial cell restitution [37]. We next assessed the impact of luteolin on TNFα-induced COX-2 gene expression in HT29 cells. Cells were treated with luteolin (100 µM) for 45 min, then treated with TNFα (5 ng/ml) for 12 h and 24 h and COX-2 mRNA and protein accumulation were determined by RT-PCR and Western blot analysis, respectively. As seen in Fig. 8, luteolin completely blocked TNFα-induced COX-2 mRNA accumulation (Fig. 8A) and protein expression (Fig. 8B) in HT29 cells. Similarly, TNFα-induced PGE_2_ secretion is blocked in luteolin-treated HT29 cells (Fig. 8C).

## Discussion

NF-κB drives the expression of a subset of target genes that have important functions in salvaging enterocytes from apoptosis and in promoting restitution in various models of gastrointestinal damage. For example, ischemia-reperfusion and radiation-induced intestinal injury is worse in mice with selective IKKβ deletion in enterocytes [45], [46]. Also, DSS-induced colitis is exacerbated in MyD88^−/−^ mice, a critical adaptor protein involved in TLR-induced NF-κB signaling [47]. Interestingly, PGE_2_-expressing stromal cells within the lamina propria reposition near the intestinal epithelial stem cell compartment following DSS-induced injury through a MyD88-dependent mechanism, a process promoting cellular proliferation and mucosal healing [48]. Noteworthy, we found that luteolin inhibits DSS-induced, NF-κB dependent enterocyte COX-2 expression in both total colonic protein extracts and purified enterocytes obtained from luteolin-fed mice. Similarly, in vitro experiments showed that luteolin blocks TNFα-induced COX-2 gene expression and PGE_2_ secretion in HT29 cells. Interestingly, luteolin attenuated restitution in a wound-healing assay in rat intestinal epithelial cells IEC-18 in vitro (69% of control, p<0.01), which was partially restored by adding exogenous PGE_2_ (87% of control, p<0.01 versus luteolin treatment alone). Thus, COX-2 and its derivative metabolites seem to play an important role in maintaining mucosal homeostasis. Of note, it should be stressed that although blocking COX-2 expression appears detrimental in the DSS model of acute colitis, a luteolin-rich diet may be beneficial in preventing colorectal cancer, where COX-2 overexpression is associated with carcinogenesis [49]–[51].

**Figure 9:**
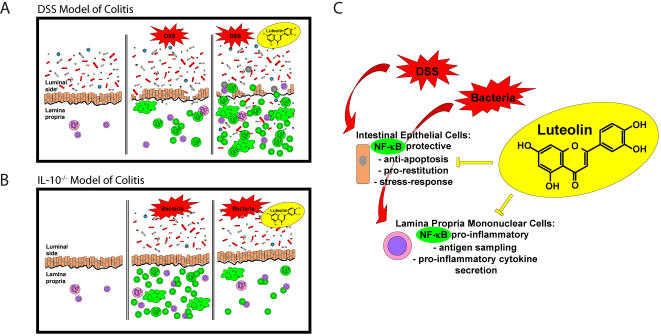
Proposed model for the differential effect of luteolin in DSS-induced acute colitis and in spontaneous chronic colitis in IL-10^−/−^-mice. DSS-induced acute colitis in NF-κB^EGFP^ transgenic mice correlates with enhanced EGFP expression (NF-κB activation) in the lamina propria due to the disruption of the epithelial monolayer and the uptake of luminal antigens by lamina propria mononuclear cells (A; middle panel). An impaired epithelial wound-healing response and increased apoptosis in luteolin-fed mice lead to enhanced EGFP expression in lamina propria mononuclear cells and exacerbate DSS-induced colitis (A; right panel). In the context of a primarily intact epithelial layer during colitis in IL-10^−/−^;NF-κB^EGFP^ mice, luteolin decreases bacteria-induced EGFP expression in lamina propria mononuclear cells, which correlates with an attenuation of colitis (B; middle and right panel). Schematic representation of luteolin's impact on intestinal epithelial cells and lamina propria mononuclear cells: NF-κB activation in intestinal epithelial cells is likely protective (wound-healing response, anti-apoptotis), and its inhibition by luteolin consequently aggravates intestinal damage. As opposed, antigen- (e.g. bacteria-) induced NF-κB activation in lamina propria mononuclear cells leads to NF-κB dependent pro-inflammatory cytokine secretion, thus its inhibition by luteolin likely ameliorates intestinal inflammation (C).

Exacerbated colitis in luteolin-treated, DSS-exposed mice may involve a higher susceptibility of enterocytes to undergo signal-induced apoptosis and a decreased ability to initiate restitution. This possibility is supported by our findings that luteolin fed mice displayed enhanced caspase 3 processing in colonic tissue following DSS exposure, suggesting a higher rate of apoptosis. In addition, in vitro experiments showed that luteolin sensitizes HT29 cells to TNFα-induced caspase-3 processing/activity as well as DNA fragmentation. These effects were associated with blockade of TNFα-induced NF-κB transcriptional activity and the concomitant decreased expression of down-stream target genes C-IAP1 and C-IAP2. The failure to maintain an intact intestinal epithelium and to repair DSS-induced intestinal epithelial damage would result in an increased uptake of luminal antigens, including bacteria and bacterial products, and thus lead to the activation of underlying immune cells and the mounting of an inflammatory response: In the DSS model of acute chemical-induced colitis, luteolin's foremost impact is likely geared towards enterocytes, inhibiting NF-κB-dependent protective molecules. Enhanced injury then leads to enhanced NF-κB activation (EGFP expression) in mononuclear cells from the colonic lamina propria, where luteolin's inhibitory effect on NF-κB in LPMNC is likely overcome by the strong impact of abundantly translocated luminal antigens. Enhanced lamina propria NF-κB activation (EGFP expression) in turn correlates with clinical and histological signs of colitis in luteolin-fed mice (Fig. 9A,C).

Numerous pro-inflammatory mediators are known to be associated with the development of DSS-induced acute inflammation including IL-6, IL-12, IL-17, IL-18 and TNFα [52]. Whether TNFα promotes intestinal injury and apoptosis in luteolin-fed DSS-treated mice remains to be formally shown. Similarly, the exact signaling events associated with enhanced COX-2 expression, NF-κB activity and caspase-3 processing in DSS-exposed mice are as of yet unclear.

Importantly, luteolin's negative impact on acute gastrointestinal damage by DSS does not impair its potential therapeutic benefits in other experimental models, for example in IL-10^−/−^ mice. In this spontaneous model of chronic, Th1-mediated intestinal inflammation, without primary epithelial damage, luteolin's inhibition of NF-κB activation (EGFP expression) is predominantly seen in lamina propria mononuclear cells (LPMNC). Its actions at least in part ameliorate the clinical and histological signs of colitis in luteolin-fed animals (Fig. 9B,C). These findings parallel our recent report demonstrating a beneficial impact of pharmacological NF-κB inhibition on colitis in IL-10^−/−^ mice [8]. Our results warrant further studies of luteolin's effects in colonic dysplasia and cancer development as well as in chronic intestinal inflammation. Since herbal compounds and their derivatives are widely available and taken by an increasing number of patients oftentimes without proper medical monitoring, our study stresses the need for further analysis of their impact on various diseases.

## References

[1] Ley RE, Peterson DA, Gordon JI (2006). Ecological and evolutionary forces shaping microbial diversity in the human intestine.. Cell.

[2] Haller D, Jobin C (2004). Interaction between resident luminal bacteria and the host: can a healthy relationship turn sour?. J Pediatr Gastroenterol Nutr.

[3] Loftus EV, Sandborn WJ (2002). Epidemiology of inflammatory bowel disease.. Gastroenterol Clin North Am.

[4] Hanauer SB (2006). Inflammatory bowel disease: epidemiology, pathogenesis, and therapeutic opportunities.. Inflamm Bowel Dis.

[5] Bouma G, Strober W (2003). The immunological and genetic basis of inflammatory bowel disease.. Nat Rev Immunol.

[6] Sartor RB (1997). Pathogenesis and immune mechanisms of chronic inflammatory bowel diseases.. Am J Gastroenterol.

[7] Cominelli F (2004). Cytokine-based therapies for Crohn's disease–new paradigms.. N Engl J Med.

[8] Karrasch T, Kim JS, Muhlbauer M, Magness ST, Jobin C (2007). Gnotobiotic IL-10−/−;NF-kappaBEGFP mice reveal the critical role of TLR/NF-kappaB signaling in commensal bacteria-induced colitis.. J Immunol.

[9] Neurath MF, Pettersson S, Meyer zum Buschenfelde KH, Strober W (1996). Local administration of antisense phosphorothioate oligonucleotides to the p65 subunit of NF-kappa B abrogates established experimental colitis in mice.. Nat Med.

[10] Jobin C, Sartor RB (2000). NF-kappaB signaling proteins as therapeutic targets for inflammatory bowel diseases.. Inflamm Bowel Dis.

[11] Hibi T, Inoue N, Ogata H, Naganuma M (2003). Introduction and overview: recent advances in the immunotherapy of inflammatory bowel disease.. J Gastroenterol.

[12] Bent S, Ko R (2004). Commonly used herbal medicines in the United States: a review.. Am J Med.

[13] Gupta RK (2004). Surge in US patents on botanicals.. Nat Biotechnol.

[14] Sampson W (2005). Studying herbal remedies.. N Engl J Med.

[15] Aggarwal BB, Shishodia S (2004). Suppression of the nuclear factor-kappaB activation pathway by spice-derived phytochemicals: reasoning for seasoning.. Ann N Y Acad Sci.

[16] Ross JA, Kasum CM (2002). Dietary flavonoids: bioavailability, metabolic effects, and safety.. Annu Rev Nutr.

[17] Manach C, Scalbert A, Morand C, Remesy C, Jimenez L (2004). Polyphenols: food sources and bioavailability.. Am J Clin Nutr.

[18] Kim JS, Jobin C (2005). The flavonoid luteolin prevents lipopolysaccharide-induced NF-kappaB signalling and gene expression by blocking IkappaB kinase activity in intestinal epithelial cells and bone-marrow derived dendritic cells.. Immunology.

[19] Hendriks JJ, Alblas J, van der Pol SM, van Tol EA, Dijkstra CD (2004). Flavonoids influence monocytic GTPase activity and are protective in experimental allergic encephalitis.. J Exp Med.

[20] Tormakangas L, Vuorela P, Saario E, Leinonen M, Saikku P (2005). In vivo treatment of acute Chlamydia pneumoniae infection with the flavonoids quercetin and luteolin and an alkyl gallate, octyl gallate, in a mouse model.. Biochem Pharmacol.

[21] Xagorari A, Papapetropoulos A, Mauromatis A, Economou M, Fotsis T (2001). Luteolin inhibits an endotoxin-stimulated phosphorylation cascade and proinflammatory cytokine production in macrophages.. J Pharmacol Exp Ther.

[22] Chen CC, Chow MP, Huang WC, Lin YC, Chang YJ (2004). Flavonoids inhibit tumor necrosis factor-alpha-induced up-regulation of intercellular adhesion molecule-1 (ICAM-1) in respiratory epithelial cells through activator protein-1 and nuclear factor-kappaB: structure-activity relationships.. Mol Pharmacol.

[23] Hougee S, Sanders A, Faber J, Graus YM, van den Berg WB (2005). Decreased pro-inflammatory cytokine production by LPS-stimulated PBMC upon in vitro incubation with the flavonoids apigenin, luteolin or chrysin, due to selective elimination of monocytes/macrophages.. Biochem Pharmacol.

[24] Ruiz PA, Haller D (2006). Functional diversity of flavonoids in the inhibition of the proinflammatory NF-kappaB, IRF, and Akt signaling pathways in murine intestinal epithelial cells.. J Nutr.

[25] Magness ST, Jijon H, Van Houten Fisher N, Sharpless NE, Brenner DA (2004). In vivo pattern of lipopolysaccharide and anti-CD3-induced NF-kappa B activation using a novel gene-targeted enhanced GFP reporter gene mouse.. J Immunol.

[26] Sugimoto K, Hanai H, Tozawa K, Aoshi T, Uchijima M (2002). Curcumin prevents and ameliorates trinitrobenzene sulfonic acid-induced colitis in mice.. Gastroenterology.

[27] Jian YT, Mai GF, Wang JD, Zhang YL, Luo RC (2005). Preventive and therapeutic effects of NF-kappaB inhibitor curcumin in rats colitis induced by trinitrobenzene sulfonic acid.. World J Gastroenterol.

[28] Okayasu I, Hatakeyama S, Yamada M, Ohkusa T, Inagaki Y (1990). A novel method in the induction of reliable experimental acute and chronic ulcerative colitis in mice.. Gastroenterology.

[29] Cooper HS, Murthy SN, Shah RS, Sedergran DJ (1993). Clinicopathologic study of dextran sulfate sodium experimental murine colitis.. Lab Invest.

[30] Williams KL, Fuller CR, Dieleman LA, DaCosta CM, Haldeman KM (2001). Enhanced survival and mucosal repair after dextran sodium sulfate-induced colitis in transgenic mice that overexpress growth hormone.. Gastroenterology.

[31] Dieleman LA, Palmen MJ, Akol H, Bloemena E, Pena AS (1998). Chronic experimental colitis induced by dextran sulphate sodium (DSS) is characterized by Th1 and Th2 cytokines.. Clin Exp Immunol.

[32] Sellon RK, Tonkonogy S, Schultz M, Dieleman LA, Grenther W (1998). Resident enteric bacteria are necessary for development of spontaneous colitis and immune system activation in interleukin-10-deficient mice.. Infect Immun.

[33] Jijon HB, Madsen KL, Walker JW, Allard B, Jobin C (2005). Serum amyloid A activates NF-kappaB and proinflammatory gene expression in human and murine intestinal epithelial cells.. Eur J Immunol.

[34] Haller D, Russo MP, Sartor RB, Jobin C (2002). IKK beta and phosphatidylinositol 3-kinase/Akt participate in non-pathogenic Gram-negative enteric bacteria-induced RelA phosphorylation and NF-kappa B activation in both primary and intestinal epithelial cell lines.. J Biol Chem.

[35] Jobin C, Haskill S, Mayer L, Panja A, Sartor RB (1997). Evidence for altered regulation of I kappa B alpha degradation in human colonic epithelial cells.. J Immunol.

[36] Cui X, Imaizumi T, Yoshida H, Tanji K, Matsumiya T (2000). Lipopolysaccharide induces the expression of cellular inhibitor of apoptosis protein-2 in human macrophages.. Biochim Biophys Acta.

[37] Karrasch T, Steinbrecher KA, Allard B, Baldwin AS, Jobin C (2006). Wound-induced p38MAPK-dependent histone H3 phosphorylation correlates with increased COX-2 expression in enterocytes.. J Cell Physiol.

[38] Ciacci C, Lind SE, Podolsky DK (1993). Transforming growth factor beta regulation of migration in wounded rat intestinal epithelial monolayers.. Gastroenterology.

[39] Egan LJ, de Lecea A, Lehrman ED, Myhre GM, Eckmann L (2003). Nuclear factor-kappa B activation promotes restitution of wounded intestinal epithelial monolayers.. Am J Physiol Cell Physiol.

[40] Morteau O, Morham SG, Sellon R, Dieleman LA, Langenbach R (2000). Impaired mucosal defense to acute colonic injury in mice lacking cyclooxygenase-1 or cyclooxygenase-2.. J Clin Invest.

[41] Fukata M, Chen A, Klepper A, Krishnareddy S, Vamadevan AS (2006). Cox-2 is regulated by Toll-like receptor-4 (TLR4) signaling: Role in proliferation and apoptosis in the intestine.. Gastroenterology.

[42] Maeda S, Yoshida H, Mitsuno Y, Hirata Y, Ogura K (2002). Analysis of apoptotic and antiapoptotic signalling pathways induced by Helicobacter pylori.. Gut.

[43] Tsurutani J, Castillo SS, Brognard J, Granville CA, Zhang C (2005). Tobacco components stimulate Akt-dependent proliferation and NFkappaB-dependent survival in lung cancer cells.. Carcinogenesis.

[44] Yan SR, Joseph RR, Rosen K, Reginato MJ, Jackson A (2005). Activation of NF-kappaB following detachment delays apoptosis in intestinal epithelial cells.. Oncogene.

[45] Egan LJ, Eckmann L, Greten FR, Chae S, Li ZW (2004). IkappaB-kinasebeta-dependent NF-kappaB activation provides radioprotection to the intestinal epithelium.. Proc Natl Acad Sci U S A.

[46] Chen LW, Egan L, Li ZW, Greten FR, Kagnoff MF (2003). The two faces of IKK and NF-kappaB inhibition: prevention of systemic inflammation but increased local injury following intestinal ischemia-reperfusion.. Nat Med.

[47] Araki A, Kanai T, Ishikura T, Makita S, Uraushihara K (2005). MyD88-deficient mice develop severe intestinal inflammation in dextran sodium sulfate colitis.. J Gastroenterol.

[48] Brown SL, Riehl TE, Walker MR, Geske MJ, Doherty JM (2007). Myd88-dependent positioning of Ptgs2-expressing stromal cells maintains colonic epithelial proliferation during injury.. J Clin Invest.

[49] Oshima M, Dinchuk JE, Kargman SL, Oshima H, Hancock B (1996). Suppression of intestinal polyposis in Apc delta716 knockout mice by inhibition of cyclooxygenase 2 (COX-2).. Cell.

[50] Kawamori T, Rao CV, Seibert K, Reddy BS (1998). Chemopreventive activity of celecoxib, a specific cyclooxygenase-2 inhibitor, against colon carcinogenesis.. Cancer Res.

[51] Wendum D, Masliah J, Trugnan G, Flejou JF (2004). Cyclooxygenase-2 and its role in colorectal cancer development.. Virchows Arch.

[52] Lorenz RG, McCracken VJ, Elson CO (2005). Animal models of intestinal inflammation: ineffective communication between coalition members.. Springer Semin Immunopathol.

